# Effectiveness of a physical literacy intervention program for young and middle-aged adults with hypertension: a mixed-methods quasi-experimental study

**DOI:** 10.3389/fendo.2026.1944805

**Published:** 2026-10-20

**Authors:** Guiyue Ma

**Affiliations:** 1 School of Nursing, Anhui University of Chinese Medicine, Hefei, Anhui, China; 2 Key Laboratory of Geriatric Nursing and Health, Anhui University of Chinese Medicine, Hefei, Anhui, China

**Keywords:** hypertension, middle aged, non-randomized controlled trials as topic, physical activity, physical literacy intervention, young adult

## Abstract

**Objective:**

To examine whether a theoretically grounded, 8-week physical literacy intervention incorporating salutogenic model components (sense of coherence, social support, and self-efficacy) could improve physical activity levels and clinical outcomes in young and middle-aged adults with Grade 1 hypertension, and to explore participants’ subjective experiences of the program.

**Methods:**

A mixed-methods quasi-experimental study was conducted between August and October 2024. Forty patients aged 18–59 years were recruited from a community healthcare center and allocated to the intervention group (n = 20) or control group (n = 20). The intervention group received a multidimensional physical-literacy program, while the control group received usual care. Five participants were lost to follow-up; 35 (intervention: n = 17; control: n = 18) completed all three assessments: baseline (T0), post-intervention (T1), and one-month follow-up (T2). Quantitative outcomes (SBP, BMI, physical-literacy scores, social support, sense of coherence, self-efficacy, and physical activity) were analyzed using independent *t*-tests or Mann-Whitney *U* tests for between-group comparisons and paired *t*-tests or Wilcoxon signed-rank tests for within-group changes, with significance set at α = 0.05 (two-tailed). Qualitative data from semi-structured interviews were analyzed using inductive content analysis.

**Results:**

Of the 40 recruited participants, 35 (87.5%) completed the study. Post-intervention, the intervention group demonstrated significant reductions in systolic blood pressure (SBP), body weight, and BMI compared to baseline (all *P* < 0.05). Significant improvements were also observed in PL scores, social support, sense of coherence, and self-efficacy (*P* < 0.05). Qualitative findings corroborated these results, highlighting enhanced health literacy and stress alleviation, despite some logistical challenges.

**Conclusion:**

The PL intervention is a feasible and effective strategy to improve health outcomes and psychosocial resilience in hypertensive adults, offering a promising approach for community-based chronic disease management.

## Introduction

Hypertension remains a paramount global public health challenge and a leading modifiable risk factor for cardiovascular disease (CVD), stroke, and premature mortality (1–3). In China, the prevalence of hypertension has been steadily rising, particularly among young and middle-aged adults, who have experienced a concerning increase in incidence over the past three decades (1). Current data indicate that young and middle-aged individuals now account for 67.5% of China’s 245 million hypertensive patients, with this demographic witnessing a staggering 95.9% rise in prevalence during the last thirty years (2). While hypertension management has traditionally focused on older populations, the burgeoning number of young and middle-aged patients has become a critical concern. This demographic not only faces heightened cardiovascular risks but also represents a primary target for early intervention due to their longer life expectancy (3). Without effective management, these patients face significant lifetime risks of adverse cardiovascular events, such as myocardial infarction and heart failure, resulting in an increased disease burden and diminished quality of life.

Several factors drive the development of hypertension in young and middle-aged adults, including sedentary lifestyles, chronic stress, unhealthy dietary habits, obesity, and excessive alcohol and tobacco consumption (4). These modifiable factors underscore the vital role of lifestyle interventions in optimizing health outcomes. Among various non-pharmacological strategies, physical activity (PA) has been identified as a cornerstone of blood pressure regulation and cardiovascular risk reduction (5, 6). However, despite these proven benefits, PA levels among young and middle-aged hypertensive patients remain alarmingly low. Evidence suggests that this group frequently fails to meet the minimum PA guidelines recommended by the World Health Organization (WHO) (7). The rise of sedentary behavior, exacerbated by technological advancements and rapid urbanization, has further complicated this issue, necessitating the development of targeted interventions to tackle this nascent yet significant health challenge. Importantly, current guidelines recommend lifestyle intervention, especially structured physical activity, as the first-line management strategy for young and middle-aged hypertensive patients (8). This is because this population can tolerate longer duration and higher intensity exercise compared with older adults, thereby achieving better blood pressure control and cardiovascular risk reduction.

Physical literacy (PL) has emerged as a transformative concept in health promotion. Defined as the motivation, confidence, physical competence, and knowledge necessary for individuals to engage in meaningful physical activities throughout the life course, PL provides a multidimensional framework for enhancing PA levels (9). The International Physical Literacy Association emphasizes the importance of PL in fostering lifelong engagement in physical activity, which is crucial for improving clinical outcomes in hypertensive patients (10). Unlike traditional fitness programs that often focus solely on isolated physical skills, PL addresses the cognitive, affective, and behavioral dimensions of movement, representing a holistic disposition that empowers individuals to make informed health decisions.

For young and middle-aged hypertensive patients, cultivating PL can yield significant health benefits. Enhanced physical literacy not only motivates regular PA but also equips patients with the requisite skills and confidence to overcome perceived barriers to exercise. This is especially pertinent for hypertension management, as PA improves cardiovascular fitness, lowers blood pressure, and reduces the risk of comorbidities (11). Furthermore, PL interventions can address psychological factors such as anxiety and depression, thereby contributing to enhanced mental resilience and overall quality of life (12).

Despite its potential, the application of PL in interventions for young and middle-aged hypertensive populations remains in its nascent stages. Most existing PL research focuses on children, adolescents, or the elderly, with minimal attention given to the unique needs of middle-aged adults (13). This gap underscores the need for innovative, age-appropriate interventions that address the specific challenges faced by this group. Current programs often target individual aspects of PL, such as knowledge or physical competence, while neglecting its integrative nature. A comprehensive approach that incorporates motivation, confidence, and contextual understanding is essential for achieving sustainable behavioral change.

The theoretical foundation of this study is rooted in Antonovsky’s salutogenic model, a health promotion framework emphasizing psychological resilience and coping resources (14). Central to this model is the sense of coherence (SOC), which comprises three components: comprehensibility, manageability, and meaningfulness (15). These elements enable individuals to perceive life challenges as understandable and manageable, fostering a proactive approach to health management (16). In the context of PL, SOC can enhance patients’ intrinsic motivation to engage in physical activity, provide tools to navigate barriers, and align their efforts with long-term health goals (17).

Social support also plays a critical role in shaping PA behaviors and enhancing PL (18). For young and middle-aged patients, a robust support network offers emotional encouragement and practical resources necessary for maintaining an active lifestyle (19). Studies have shown that individuals receiving adequate social support are more likely to adhere to health-promoting behaviors and achieve superior health outcomes (20). Therefore, incorporating social support into PL interventions can amplify their impact, creating a synergistic effect on individual health.

Self-efficacy, or an individual’s belief in their ability to perform specific tasks, is another key determinant of health behaviors (21). For hypertensive patients, high self-efficacy leads to greater confidence in managing their condition and sustaining PA regimens (22). PL interventions that build self-efficacy empower patients to take ownership of their health and maintain long-term adherence. Additionally, self-efficacy interacts dynamically with SOC and social support, creating a positive feedback loop that reinforces resilience and consistency in behavior (18).

Existing PL interventions have shown promising outcomes in various populations (23). For instance, school-based programs have successfully enhanced PL among students through experiential learning (24), while community-based initiatives for older adults have utilized peer support to improve PA levels (25). A recent systematic review and meta-analysis published in Sports Medicine evaluated 48 eligible physical literacy interventions and confirmed their effectiveness across five key outcome categories. The analysis revealed that these interventions produced significant positive effects, with the strongest impact observed in physical competence. This was followed by significant improvements in total physical literacy, knowledge and understanding, physical activity behavior, and motivation and confidence (26). These precedents highlight the feasibility of PL-focused approaches, providing a foundation for targeted programs for hypertensive adults (27).

This study aims to address the critical gap in PL research for young and middle-aged hypertensive patients. By developing and evaluating a tailored PL intervention, this study seeks to enhance participants’ motivation, confidence, and physical competence, thereby promoting sustained PA engagement. The intervention incorporates key elements of the salutogenic model, emphasizing SOC, social support, and self-efficacy to create a holistic and sustainable framework. Through a quasi-experimental design, the study will evaluate the feasibility and preliminary effects of the intervention, providing valuable insights for broader clinical application.

## Methods

### Study design and setting

This mixed-methods quasi-experimental study used a two-group pre-test–post-test design. It was conducted at a community healthcare center in Hefei, China, between August and October 2024.

### Study population

Participants were recruited by convenience sampling. Inclusion criteria were: (1) age 18–59 years; (2) diagnosis of Grade 1 hypertension (SBP 140–159 mmHg and/or DBP 90–99 mmHg on three separate occasions without antihypertensive medication) according to the National Guidelines for Prevention and Management of Hypertension in Primary Care (2020 Edition); and (3) ability to communicate clearly. Exclusion criteria included secondary or white-coat hypertension; severe cardiovascular, cerebrovascular, or neoplastic comorbidities; pregnancy or lactation; and concurrent participation in other clinical trials.

### Recruitment and data collection

To ensure sufficient statistical power for this pilot evaluation, a pragmatic sample size was determined based on established methodological recommendations for pilot studies (typically 20–30 participants per group). Anticipating a 20% attrition rate, with 20 participants in the intervention group and 20 in the control group.

#### Allocation

As this was a quasi-experimental study rather than a randomized controlled trial, participants were allocated using a non-randomized method based on the order of enrollment. The first 20 eligible participants who provided informed consent were assigned to the intervention group, and the subsequent 20 participants were assigned to the control group.

#### Data were collected at three time points

Baseline (T0, before the intervention) and post-intervention (T1, after the 8-week program), and one-month follow-up (T2, 1 month after the intervention). At each time point, trained research assistants administered the questionnaires in a quiet room at the community healthcare center, and clinical measurements were taken by a trained nurse. Blood pressure was measured three times with a calibrated sphygmomanometer after 5 minutes of rest, and the average was recorded. Participants who failed to complete all three assessment time-points (T0, T1 and T2) were excluded from quantitative analysis under the complete-case analysis approach. Post-intervention, semi-structured interviews (20–30 min) were conducted with 10 purposively selected participants who completed the program. Interviews were audio-recorded, transcribed verbatim within 24 hours, and supplemented with reflexive field notes.

#### Intervention protocol (TIDieR alignment)

The intervention was developed using the ADDIE (Analysis, Design, Development, Implementation, and Evaluation) model, integrating a literature review, expert Delphi consultations, and focus group insights. This multi-component physical literacy-based intervention is a tailored de-novo program specifically designed for young- and middle-aged adults with grade 1 hypertension rather than a pre-existing standardized intervention. Individual constituent techniques (e.g., SMART goal-setting, mindfulness-based stress reduction, Tai Chi, Baduanjin) are well-established evidence-based components from previous chronic-disease and physical activity promotion research. Iterative revisions to session objectives, content and delivery format were completed based on expert consensus and patient feedback. The full week-by-week implementation schedule of the final intervention protocol is presented in **Supplementary File 1**. The 8-week PLI program focused on enhancing the cognitive, affective, and physical domains of movement. Key components included:

#### Cognitive-educational workshops

Utilizing SMART goal-setting strategies to foster health literacy and personalized physical activity (PA) planning.

#### Physical competence training

Tailored exercises including strength, flexibility, and balance training, alongside traditional Chinese modalities (Tai Chi, Baduanjin) and aerobic dance, aiming for 150–300 minutes of moderate-intensity PA per week.

#### Psychosocial and digital support

Leveraging a WeChat-based peer support network and AI health assistants for 24/7 monitoring and real-time feedback.

#### Affective engagement

Implementing mindfulness-based stress reduction and reflective journaling to enhance self-efficacy and sense of coherence (SOC).

#### Intervention fidelity and adherence

Intervention fidelity was ensured through three strategies. First, all intervention sessions were delivered by a trained multidisciplinary team (nursing experts, a sports science teacher, a psychological counselor, and community health workers) who had completed a standardized 2-day training workshop covering the study background, the salutogenic theoretical framework, the intervention protocol (exercise type, intensity control, and psychological interview techniques), hypertension management guidelines, and traditional Chinese medicine (TCM) theory and traditional exercise techniques. Second, a detailed intervention manual and a physical literacy guidance handbook were developed to standardize each intervention component, with designated team leaders supervising implementation. Third, the research team held weekly online meetings to review progress, discuss problems, and adjust the protocol as needed; intervention records (text, audio, and video) were archived by a designated staff member for ongoing monitoring. Intervention adherence was assessed using session attendance records. Overall, 35 of 40 participants (87.5%) completed the 8-week intervention, with a mean course participation rate of 79.4% across the eight sessions. No adverse events were reported (0.0%).

### Outcome measures and data collection

#### Study instruments

##### Quantitative instruments

A researcher-developed demographic questionnaire, guided by expert input, captured socio-demographic variables (gender, age, marital status, education, occupation, household income, residence, living conditions, and medical insurance) and clinical data (smoking and alcohol history, disease duration, and comorbidities). Height and weight were measured to two decimal places (0.01 m; 0.01 kg), and BMI was calculated as weight (kg)/height (m)². Participants were categorized as underweight, normal weight, overweight, or obese according to Chinese adult standards.

###### Physical literacy scale for young and middle-aged patients with hypertension

This scale was developed by the research team (28). It consists of 4 dimensions and 18 items: cognitive dimension (5 items), affective dimension (3 items), physical competence dimension (5 items), and behavioral dimension (5 items). A 5-point Likert scale is used (1 = strongly disagree, 5 = strongly agree). The total score ranges from 18 to 90; higher scores indicate higher physical literacy. The scale has good reliability and validity.

###### Perceived social support scale

The Chinese version was revised by Jiang Qianjin based on Zimet’s original scale (29). It includes 3 dimensions and 12 items: family support (4 items), friend support (4 items), and other support (4 items). A 7-point Likert scale is adopted. Total scores range from 12 to 84; higher scores represent stronger perceived social support. The Cronbach’s α coefficient was 0.84 in previous studies and 0.949 in this study, showing excellent reliability.

###### Sense of coherence scale

The scale was developed by Antonovsky (30) and translated into Chinese by Bao Leiping (31). It contains 3 dimensions and 13 items: comprehensibility (5 items), manageability (4 items), and meaningfulness (4 items). A 7-point Likert scale is used, with 5 reverse-scored items. Total scores range from 13 to 91; higher scores reflect a stronger sense of coherence. The Cronbach’s α coefficient was 0.89 in previous studies and 0.849 in this study.

###### General self-efficacy scale

The Chinese version was revised by Wang Caikang (32). It is a single-dimensional scale with 10 items. A 4-point Likert scale is used (1 = completely inconsistent, 4 = completely consistent). Total scores range from 10 to 40; higher scores indicate stronger self-efficacy. The Cronbach’s α coefficient was 0.87 in previous studies and 0.917 in this study.

###### International physical activity questionnaire short form

IPAQ-SF was developed by an international consensus group in 2001 (33). The Chinese version was verified by Qu Ningning with good reliability and validity. It includes 7 items assessing walking, moderate, vigorous physical activity, and sedentary time. Physical activity level is calculated using metabolic equivalents (METs). The scale is widely used to evaluate physical activity levels.

##### Qualitative exploration

Post-intervention, semi-structured interviews (20–30 minutes) were conducted with participants to capture their perceptions of the PLI. Purposive sampling was used to select interviewees based on age, sex, and intervention adherence, ensuring variation across demographic and behavioral characteristics. Interviews were audio-recorded, transcribed verbatim within 24 hours, and supplemented with reflexive field notes. Data saturation was determined when no new codes emerged from three consecutive interviews; this point was reached after the 10th interview. The interview guide (see **Supplementary File 2**) covered four domains: overall intervention experience, perceived changes in physical literacy, facilitators and barriers to participation, and suggestions for improvement.

### Ethical approval

The study was approved by the Ethics Committee of Zhejiang Chinese Medical University (No. 20240315) and prospectively registered in the Chinese Clinical Trial Registry (ChiCTR2400086313). All procedures followed the Declaration of Helsinki, and written informed consent was obtained from all participants.

### Data analysis

Quantitative data were analyzed using SPSS 27.0. Normality was tested with the Shapiro-Wilk test. Continuous variables are presented as mean ± SD or median (IQR); between-group comparisons used independent *t*-tests or Mann-Whitney *U* tests; within-group changes used paired t-tests or Wilcoxon signed-rank tests. Categorical variables used Chi-square or Fisher’s exact tests. Significance was set at α = 0.05 (two-tailed). As this was a pilot study with a limited sample size, participants were analyzed as a single cohort.

For missing data, complete-case analysis was performed: only data from 35 participants who completed all assessment time-points (T0, T1 and T2) were included in quantitative analyses. Data from five participants lost-to-follow-up were excluded entirely, as partial follow-up records were not utilized in the present analysis. This approach assumes that data are missing completely at random (MCAR), which is noted as one limitation of this pilot quasi-experimental study.

Qualitative data were analyzed using traditional content analysis in NVivo 12.0. Transcripts were verbatim transcribed within 24 hours of each interview, retaining paralinguistic details such as filler words, pauses, and repeated phrases, and reflexive notes were written after each interview. The analysis followed four steps: (1) reading all transcripts to establish item selection criteria focused on satisfaction, gains, difficulties, and suggestions for improvement; (2) extracting meaningful statements as semantic units and comparing their similarities and differences to form preliminary items; (3) integrating items through cross-checking and team discussion to ensure coding consistency, with disagreements resolved by returning to the original transcripts or conducting supplementary interviews; and (4) refining conclusions by interpreting the underlying reasons for participants’ experiences. The researcher and a trained graduate student conducted the analysis jointly.

## Results

### Participant flow and baseline characteristics

Three participants withdrew from the intervention group: two due to work-related commitments and one following medical advice related to a foot injury. Two participants withdrew from the control group for personal reasons, including relocation and scheduling conflicts. The final analysis included 35 participants (intervention group: n = 17; control group: n = 18). Therefore, five participants who withdrew from the study were excluded from all quantitative analyses, and only data from the 35 participants with complete data across all three time-points were analyzed. As summarized in **Table 1**, no statistically significant differences were observed between the intervention and control groups in socio-demographic variables or baseline clinical characteristics (all *P* > 0.05), confirming adequate baseline comparability between the two groups.

**Table 1:** Baseline socio-demographic and clinical characteristics of participants.

Variable	Group	Intervention [n=17, n (%)/( $\bar{x}$ ± *s*)]	Control [n=18, n (%)/( $\bar{x}$ ± *s*)]	*t/χ^2^*	*P*
Age (years)		43.12 ± 7.32	41.61 ± 7.85	0.586^a^	0.562
Gender	Male	6 (35.3)	9 (50.0)	0.772^b^	0.380
	Female	11 (64.7)	9 (50.0)		
Marital status	Unmarried	2 (11.8)	5 (27.8)	1.401^b^	0.237
	Married	15 (88.2)	13 (72.2)		
Education level	Junior high school and below	3 (17.6)	2 (11.1)	1.067^b^	0.785
	High school	7 (41.2)	6 (33.3)		
	Junior college	4 (23.5)	7 (38.9)		
	Bachelor’s degree and above	3 (17.6)	3 (16.7)		
Occupation	Unemployed	3 (17.6)	2 (11.1)	0.305^b^	0.581
	Employed	14 (82.4)	16 (88.9)		
Monthly household income	<1000	0 (0.0)	0 (0.0)	0.563^b^	0.755
	1000∼3000	3 (17.6)	5 (27.8)		
	3001∼5000	8 (47.1)	8 (44.4)		
	>5000	6 (35.3)	5 (27.8)		
Residential location	Urban	14 (82.4)	13 (72.2)	0.509^b^	0.476
	Rural	3 (17.6)	5 (27.8)		
Living situation	Living with family	16 (94.1)	14 (77.8)	1.906^b^	0.167
	Living alone	1 (5.9)	4 (22.2)		
Insurance type	Public or social medical insurance	8 (47.1)	11 (61.1)	1.513^b^	0.469
	New rural cooperative medical system	8 (47.1)	7 (38.9)		
	Self-paid or others	1 (5.9)	0 (0.0)		
Smoking history	Never smoked	13 (76.5)	9 (50.0)	2.901^b^	0.234
	Formerly smoked, now quit	2 (11.8)	3 (16.7)		
	Currently smoking	2 (11.8)	6 (33.3)		
Drinking history	Never drank	10 (58.8)	11 (61.1)	2.821^b^	0.244
	Formerly drank, now quit	4 (23.5)	1 (5.6)		
	Currently drinking	3 (17.6)	6 (33.3)		
Disease duration	1∼5	10 (58.8)	8 (44.4)	12.181^b^	0.592
	6∼10	4 (23.5)	8 (44.4)		
	11∼15	2 (11.8)	1 (5.6)		
	16∼20	1 (5.9)	1 (5.6)		
Comorbidities	No	11 (64.7)	8 (44.4)	1.446^b^	0.229
	Yes	6 (35.3)	10 (55.6)		

^a^ Independent-samples t-test.

^b^ Chi-square test.

### Pre- and post-intervention objective indicators

Significant group-by-time interaction effects were detected for body weight, BMI and systolic blood pressure (SBP, all *P* < 0.001, **Tables 2**, **3**). At the one-month follow-up after intervention, participants in the intervention group exhibited significantly lower body weight, BMI and systolic blood pressure compared with the control group (*P*< 0.05). Although diastolic blood pressure showed a statistically significant group-by-time interaction effect (*P* = 0.005), pairwise between-group comparisons at individual post-intervention and follow-up time-points revealed no statistically significant differences in DBP between groups.

**Table 2:** Within-group comparisons of objective indicators before and after intervention( $\bar{x}$ ± *s*).

Variable	Group	T0 (Baseline)	T1 (Post-intervention)	T2 (one-month follow-up)
Body weight (kg)	Intervention (n=17)	69.76 ± 10.80	67.01 ± 10.67	65.56 ± 10.61
	Control (n=18)	72.38 ± 14.04	72.45 ± 12.47	73.32 ± 11.76
	*t*	-0.616	-1.384	-2.046
	*P*	0.542	0.176	0.049
	Mean difference (95% CI)	-2.619 (-11.269, 6.031)	-5.444 (-13.449, 2.561)	-7.757 (-15.473, -0.042)
	Cohen’s d	-0.208	-0.468	-0.692
BMI(kg/m^2^)	Intervention (n=17)	25.16 ± 2.13	24.16 ± 2.15	23.63 ± 2.09
	Control (n=18)	24.61 ± 1.63	24.70 ± 1.23	25.03 ± 0.96
	*t/Z*	0.870	-0.903	-2.522
	*P*	0.391	0.375	0.019
	Mean difference (95% CI)	0.556 (-0.744, 1.856)	-0.539 (-1.738, 0.659)	-1.399 (-2.550, -0.249)
	Cohen’s d	0.294	-0.310	-0.859
SBP (mmHg)	Intervention (n=17)	150.24 ± 5.85	144.00 ± 6.26	142.88 ± 5.73
	Control (n=18)	149.78 ± 4.11	150.39 ± 4.63	151.28 ± 3.53
	*t*	0.266	-3.419	-5.182
	*P*	0.792	0.002	<0.001
	Mean difference (95% CI)	0.458 (-3.002, 3.917)	-6.389 (-10.158, -2.619)	-8.395 (-11.724, -5.067)
	Cohen’s d	0.091	-1.166	-1.776
DBP(mmHg)	Intervention (n=17)	95.53 ± 2.67	93.94 ± 1.56	91.88 ± 2.57
	Control (n=18)	94.17 ± 2.31	93.78 ± 1.66	93.50 ± 2.18
	*t*	1.618	0.299	-2.013
	*P*	0.115	0.767	0.052
	Mean difference (95% CI)	1.363 (-0.351, 3.077)	0.164 (-0.947, 1.275)	-1.618 (-3.253, 0.017)
	Cohen’s d	0.547	0.101	-0.681

**Table 3:** Repeated-measures ANOVA for objective indicators of participants.

Variable	Group effect *F*(*P*)	Time effect *F*(*P*)	Group × Time *F*(*P*)	η²p (interaction)
Body weight (kg)	1.754 (0.194)	15.615 (<0.001)	34.086 (<0.001)	0.508
BMI (kg/m^2^)	0.635 (0.431)	14.996 (<0.001)	41.071 (<0.001)	0.554
SBP (mmHg)	8.424 (0.007)	29.034 (<0.001)	56.920 (<0.001)	0.633
DBP (mmHg)	0.003 (0.955)	11.787 (<0.001)	5.685 (0.005)	0.147

η²p = partial eta-squared for interaction effect.

### Physical literacy levels

Significant group-by-time interactions were detected for total score and all four sub-dimensions (cognition, emotion, physical competence, behavior, all *P* < 0.05, **Tables 4**, **5**). The intervention group obtained markedly higher total physical-literacy scores and sub-dimension scores at post-intervention and one-month follow-up relative to the control group (all *P* < 0.001).

**Table 4:** Comparison of physical-literacy levels between the two groups (score, $\bar{x}$ ± *s*).

Variable	Group	T0 (Baseline)	T1 (Post-intervention)	T2 (one-month follow-up)
Total scores	Intervention (n=17)	60.65 ± 5.16	75.00 ± 3.26	73.59 ± 4.39
	Control(n=18)	62.33 ± 7.90	63.11 ± 4.85	62.44 ± 5.14
	*t*	-0.743	8.460	6.882
	*P*	0.463	<0.001	<0.001
	Mean difference (95% CI)	-1.686 (-6.306, 2.933)	11.889 (9.029, 14.749)	11.144 (7.849, 14.438)
	Cohen’s d	-0.251	2.861	2.327
Cognition	Intervention (n=17)	17.24 ± 1.95	21.59 ± 1.18	21.59 ± 1.28
	Control(n=18)	18.22 ± 1.90	18.39 ± 2.00	18.06 ± 1.80
	*t*	-1.517	5.798	6.665
	*P*	0.139	<0.001	<0.001
	Mean difference (95% CI)	-0.987 (-2.310, 0.337)	3.199 (2.060, 4.338)	3.533 (2.454, 4.611)
	Cohen’s d	-0.513	1.933	2.254
Emotion	Intervention (n=17)	10.71 ± 1.57	12.41 ± 1.18	11.71 ± 1.61
	Control(n=18)	10.22 ± 1.90	10.17 ± 0.99	10.67 ± 1.46
	*t*	0.819	6.137	2.005
	*P*	0.419	<0.001	0.053
	Mean difference (95% CI)	0.484 (-0.718, 1.685)	2.245 (1.500, 2.989)	1.039 (-0.015, 2.094)
	Cohen’s d	0.277	2.075	0.678
Physical competence	Intervention (n=17)	16.76 ± 2.86	20.06 ± 1.92	19.41 ± 1.80
	Control(n=18)	16.61 ± 4.49	17.33 ± 2.30	16.89 ± 2.35
	*t*	0.120	3.793	3.548
	*P*	0.905	<0.001	0.001
	Mean difference (95% CI)	0.154 (-2.452, 2.760)	2.725 (1.263, 4.187)	2.523 (1.076, 3.970)
	Cohen’s d	0.041	1.283	1.200
Behavior	Intervention (n=17)	15.94 ± 2.49	20.94 ± 1.30	20.88 ± 1.65
	Control(n=18)	17.28 ± 1.45	17.22 ± 1.35	16.83 ± 2.15
	*t*	-1.957	8.291	6.220
	*P*	0.059	<0.001	<0.001
	Mean difference (95% CI)	-1.337 (-2.726, 0.053)	3.719 (2.807, 4.632)	4.049 (2.725, 5.374)
	Cohen’s d	-0.662	2.804	2.104

Parametric indices (mean difference, Cohen’s d) were reported for normally-distributed variables.

**Table 5:** Repeated-measures ANOVA for physical-literacy scores.

Variable	Group effect *F*(*P*)	Time effect *F*(*P*)	Group × Time *F*(*P*)	η²p (interaction)
Total scores	22.279 (<0.001)	45.814 (<0.001)	39.665 (<0.001)	0.546
Cognition	15.506 (<0.001)	41.166 (<0.001)	41.166 (<0.001)	0.555
Emotion	13.154 (<0.001)	4.130 (0.032)	4.142 (0.032)	0.112
Physical competence	4.902 (0.034)	12.720 (<0.001)	6.031 (0.013)	0.155
Behavior	24.302 (<0.001)	28.542 (<0.001)	34.779 (<0.001)	0.513

η²p = partial eta-squared for interaction effect.

### Social support levels

The total social-support score showed significant group-by-time interaction (*P* < 0.001, **Tables 6**, **7**). Significant inter-group differences in total social-support, family support and other support were found at post-intervention and one-month follow-up (*P* < 0.05). By contrast, friend-support showed no significant between-group differences across any assessment time-points.

**Table 6:** Comparison of social-support levels between the two groups [score, $\bar{x}$ ± s/M(*P*25, *P*75)].

Variable	Group	T0 (Baseline)	T1 (Post-intervention)	T2 (one-month follow-up)
Total scores	Intervention (n=17)	60.88 ± 12.94	74.35 ± 4.46	67.59 ± 5.62
	Control (n=18)	60.78 ± 10.74	61.72 ± 9.20	58.72 ± 8.61
	*t*	0.026	5.215	3.626
	*P*	0.979	<0.001	0.001
	Mean difference (95% CI)	0.105 (-8.053, 8.263)	12.631 (7.612, 17.650)	8.861 (3.833, 13.890)
	Cohen’s d	0.009	1.732	1.212
Family support	Intervention (n=17)	23.00 (16.00, 24.00)	26.00 (25.00, 27.00)	24.06 ± 1.75
	Control (n=18)	21.50 (16.00, 28.00)	22.50 (16.00, 24.00)	20.39 ± 3.57
	*t/Z*	-0.235	-4.442	3.897
	*P*	0.814	<0.001	<0.001
	*r*	0.046	0.873	0.582
Friend support	Intervention (n=17)	19.00 (16.00, 24.00)	24.00 (21.00, 25.50)	22.00 (19.00, 23.50)
	Control (n=18)	19.50 (16.00, 24.50)	20.50 (16.00, 25.00)	21.00 (17.00, 24.00)
	*t/Z*	-0.235	-1.582	-1.508
	*P*	0.815	0.114	0.132
	*r*	0.046	0.310	0.297
Other support	Intervention (n=17)	20.00 (16.00, 22.50)	25.18 ± 1.47	22.29 ± 2.14
	Control (n=18)	18.00 (17.00, 20.25)	19.83 ± 2.09	19.28 ± 2.19
	*t/Z*	-0.533	8.694	4.113
	*P*	0.594	<0.001	<0.001
	*r*	0.105	0.974	0.624

Parametric indices (mean difference, Cohen’s d) were reported for normally-distributed variables. Non-normally-distributed variables were analyzed by Mann-Whitney *U* test, with non-parametric effect-size *r.*

**Table 7:** Repeated-measures ANOVA and generalized estimating equations for social-support scores.

Variable	Group effect *F/*Wald χ^2^(*P*)	Time effect *F/*Wald χ^2^(*P*)	Group × Time *F/*Wald χ^2^(*P*)	η²p (interaction)
Total scores^a^	22.279 (<0.001)	45.814 (<0.001)	39.665 (<0.001)	0.321
Family support^b^	7.417 (0.006)	36.124 (<0.001)	21.948 (<0.001)	—
Friend support^b^	1.037 (0.309)	32.365 (<0.001)	9.455 (0.009)	—
Other support^b^	23.454 (<0.001)	71.607 (<0.001)	34.255 (<0.001)	—

^a^Analyzed using repeated measures ANOVA; *F* values are reported, and η²p is reported as the effect size.

^b^Analyzed using generalized estimating equations (GEE); Wald χ² statistics are reported. η²p is not applicable to GEE models. “—” indicates not applicable.

### Sense of coherence levels

There was a significant group-by-time interaction for the total sense-of-coherence score and most sub-domains (**Tables 8**, **9**). Total sense-of-coherence scores were significantly higher in the intervention group at post-intervention and follow-up (*P* < 0.001). Notably, the manageability sub-scale showed no significant group-by-time interaction (*P*>0.05), indicating no differential change in this dimension between groups.

**Table 8:** Comparison of sense of coherence levels between the two groups [score, $\bar{x}$ ± s/M(*P*25, *P*75)].

Variable	Group	T0 (Baseline)	T1 (Post-intervention)	T2 (one-month follow-up)
Total scores	Intervention (n=17)	60.00 (44.00, 70.00)	72.59 ± 5.27	69.82 ± 4.41
	Control (n=18)	55.00 (52.00, 64.00)	58.72 ± 6.49	57.00 ± 5.90
	*t/Z*	-0.017	6.916	7.251
	*P*	0.987	<0.001	<0.001
	*r*	0.003	0.892	0.899
Comprehensibility	Intervention (n=17)	18.00 (15.00, 28.00)	27.82 ± 3.81	26.59 ± 3.24
	Control (n=18)	20.00 (16.00, 26.00)	20.67 ± 4.34	20.11 ± 3.41
	*t/Z*	-0.066	5.172	5.752
	*P*	0.947	<0.001	<0.001
	*r*	0.013	0.794	0.846
Manageability	Intervention (n=17)	19.88 ± 6.40	21.24 ± 2.08	21.47 ± 1.91
	Control (n=18)	20.39 ± 5.72	20.06 ± 2.73	19.33 ± 2.40
	*t*	-0.247	1.431	2.904
	*P*	0.806	0.162	0.007
	Mean difference (95% CI)	-0.506 (-4.676, 3.663)	1.179 (-0.497, 2.856)	2.137 (0.640, 3.634)
	Cohen’s d	-0.084	0.484	0.982
Meaningfulness	Intervention (n=17)	17.71 ± 3.87	23.53 ± 1.84	22.00 (20.50, 24.00)
	Control (n=18)	18.06 ± 3.51	18.00 ± 2.47	17.00 (16.00, 20.00)
	*t/Z*	-0.280	7.466	-3.888
	*P*	0.781	<0.001	<0.001
	*r*	0.039	0.892	0.765

Parametric indices (mean difference, Cohen’s d) were reported for normally-distributed variables. Non-normally-distributed variables were analysed by Mann-Whitney *U* test, with non-parametric effect-size*r*.

**Table 9:** Repeated-measures ANOVA and generalized estimating equations for sense of coherence scores.

Variable	Group effect *F/*Wald χ^2^ (*P*)	Time effect *F/*Wald χ^2^ (*P*)	Group × Time *F/*Wald χ^2^ (*P*)	η²p (interaction)
Total scores^b^	12.416 (<0.001)	45.845 (<0.001)	28.022 (<0.001)	—
Comprehensibility^b^	8.675 (0.003)	29.519 (<0.001)	18.374 (<0.001)	—
Manageability^a^	0.867 (0.358)	0.218 (0.676)	1.502 (0.231)	0.044
Meaningfulness^b^	16.603 (<0.001)	52.592 (<0.001)	62.635 (<0.001)	—

^a^Analyzed using repeated measures ANOVA; *F* values are reported, and η²p is reported as the effect size.

^b^Analyzed using generalized estimating equations (GEE); Wald χ² statistics are reported. η²p is not applicable to GEE models. “—” indicates not applicable.

### General self-efficacy levels

General self-efficacy presented a significant group-by-time interaction effect (*P*< 0.001, **Tables 10**, **11**). Self-efficacy was significantly higher in the intervention group at both post-intervention and one-month follow-up.

**Table 10:** Comparison of general self-efficacy levels between the two groups (score, $\bar{x}$ ± *s*/M(*P*25, *P*75)).

Variable	Group	T0 (Baseline)	T1 (Post-intervention)	T2 (one-month follow-up)
Total scores	Intervention (n=17)	27.00 (22.00, 31.50)	36.65 ± 2.69	35.06 ± 2.49
	Control (n=18)	28.50 (22.25, 40.00)	32.06 ± 5.64	31.78 ± 4.07
	*t/Z*	-0.979	3.100	2.897
	*P*	0.328	0.005	0.007
	*r*	0.193	0.477	0.503

Non-normally-distributed variable was analysed by Mann-Whitney *U* test, with non-parametric effect-size *r*.

**Table 11:** Generalized linear model analysis of self-efficacy scores between the two groups of patients.

Variable	Group effect *F/*Wald χ^2^(*P*)	Time effect *F/*Wald χ^2^(*P*)	Group × Time *F/*Wald χ^2^(*P*)	η²p (interaction)
Total scores	1.259 (0.262)	30.779 (<0.001)	18.550 (<0.001)	—

“—” indicates not applicable.

### Physical activity levels

In terms of physical activity outcomes, significant group-by-time interactions were identified for weekly total energy expenditure, moderate-intensity exercise, walking time and sedentary time (*P* < 0.05, **Tables 12**, **13**). The intervention group achieved greater weekly total energy expenditure, moderate-intensity exercise and walking time. No significant group-by-time interaction was found for high-intensity exercise (*P*>0.05). While sedentary time decreased in the intervention group without reaching statistical significance.

**Table 12:** Comparison of physical activity levels between the two groups [score, $\bar{x}$ ± s/M(*P*25, *P*75)].

Variable	Group	T0 (Baseline)	T1 (Post-intervention)	T2 (one-month follow-up)
Weekly total energy expenditure (min)	Intervention (n=17)	1161.74 ± 280.73	1622.12 ± 272.13	1584.00 (1357.50, 1845.00)
	Control (n=18)	1132.64 ± 270.48	1261.86 ± 208.23	1213.50 (969.38, 1382.38)
	*t/Z*	0.312	4.414	-3.664
	*P*	0.757	<0.001	<0.001
	*r*	0.049	0.690	0.725
High-intensity exercise (min)	Intervention (n=17)	64.41 ± 26.21	75.00 (60.00, 95.00)	75.00 ± 20.62
	Control (n=18)	54.17 ± 19.80	60.00 (45.00, 62.50)	65.56 ± 21.62
	*t/Z*	1.310	-3.064	1.321
	*P*	0.199	0.002	0.196
	*r*	0.216	0.582	0.242
Moderate-intensity exercise (min)	Intervention (n=17)	102.65 ± 45.86	135.88 ± 28.68	125.00 (100.00, 167.50)
	Control (n=18)	98.06 ± 39.56	113.61 ± 33.60	85.00 (67.50, 108.75)
	*t/Z*	0.318	2.103	-2.937
	*P*	0.753	0.043	0.003
	*r*	0.042	0.346	0.578
Walking (min)	Intervention (n=17)	71.47 ± 32.82	125.00 (110.00, 150.00)	140.29 ± 35.33
	Control (n=18)	93.06 ± 33.57	112.50 (75.00, 125.00)	92.78 ± 29.32
	*t/Z*	-1.922	-2.287	4.340
	*P*	0.063	0.022	<0.001
	*r*	0.389	0.448	0.690
Sedentary time (min)	Intervention (n=17)	409.41 ± 70.49	368.24 ± 52.35	353.53 ± 46.90
	Control (n=18)	390.00 ± 75.85	370.56 ± 65.66	391.11 ± 62.77
	*t*	0.783	-0.115	-1.997
	*P*	0.439	0.909	0.054
	Mean difference (95% CI)	19.412 (-24.789, 69.846)	-2.320 (-20.149, 38.674)	-37.582 (-75.867, 0.703)
	Cohen’s d	0.265	-0.039	-0.675

Non-normally-distributed variables were analysed by Mann-Whitney *U* test, with non-parametric effect-size*r*.

**Table 13:** Repeated-measures ANOVA and generalized estimating equations for physical activity indicators.

Variable	Group effect *F/*Wald χ^2^(*P*)	Time effect *F/*Wald χ^2^(*P*)	Group × Time *F/*Wald χ^2^(*P*)	η²p (interaction)
Weekly total energy expenditure(min)^b^	21.643 (<0.001)	32.679 (<0.001)	20.097 (<0.001)	—
High-intensity exercise(min)^b^	8.717 (0.003)	2.592 (0.274)	2.351 (0.309)	—
Moderate-intensity exercise(min)^b^	7.147 (0.008)	6.540 (0.038)	6.811 (0.033)	—
Walking(min)^b^	5.661 (0.017)	52.178 (<0.001)	35.199 (<0.001)	—
Sedentary time(min)^a^	0.110 (0.742)	22.051 (<0.001)	16.316 (<0.001)	0.331

^a^Analyzed using repeated measures ANOVA; *F* values are reported, and η²p is reported as the effect size.

^b^Analyzed using generalized estimating equations (GEE).

### Feasibility and degree of satisfaction

The overall study completion rate was 87.5% (35/40), dropout rate 12.5% (5/40), and no adverse events occurred. The intervention-group completion rate was 85% (17/20), and the control-group completion rate was 90% (18/20). Overall, participants reported high satisfaction with the intervention. Specifically, 88.2% expressed general satisfaction, and 76.5% found the program content clear. Additionally, 64.7% considered the assigned homework and practice time to be appropriate. Regarding delivery methods, 52.9% were satisfied, while 35.3% rated them as average, indicating potential areas for optimization. The intervention was generally feasible and well-received, with no adverse effects reported during or after the study activities, supporting its suitability for young and middle-aged patients with hypertension. Detailed results are provided in **Table 14**.

**Table 14:** Satisfaction of participants with the study.

Variables		Frequency	Percentage (%)
Overall satisfaction	Satisfied	15	88.2
	Neutral	2	11.8
	Dissatisfied	0	0.0
	Not applicable	0	0.0
Clarity of course content	Good	13	76.5
	Neutral	3	17.6
	Poor	0	0.0
	Not applicable	1	5.9
Appropriateness of homework and practice time	Satisfied	11	64.7
	Neutral	3	17.6
	Dissatisfied	1	5.9
	Not applicable	2	11.8
Content delivery method	Satisfied	9	52.9
	Neutral	6	35.3
	Unsatisfied	1	5.9
	Not applicable	1	5.9

### Qualitative research results

#### General information of interviewees

Ten participants aged 32-55 years took part in semi-structured interviews; their demographic characteristics are summarized in **Table 15**.

**Table 15:** General information of interviewees (n=10).

Number	Gender	Age	Marital status	Education level	Occupation	Per capita monthly household income	Home residence
P1	Male	55	Married	High school	Employed	3001-5000	Urban
P2	Female	50	Married	Junior high school and below	Unemployed	>5000	Urban
P3	Male	40	Married	High school	Employed	3001-5000	Urban
P4	Female	32	Unmarried	Junior college	Employed	3001-5000	Urban
P5	Female	46	Married	High school	Employed	>5000	Rural
P6	Male	50	Married	High school	Employed	3001-5000	Urban
P7	Female	36	Married	Junior college	Employed	1000-3000	Urban
P8	Female	44	Married	Junior high school and below	Employed	>5000	Urban
P9	Male	52	Married	High school	Employed	3001-5000	Urban
P10	Female	40	Married	Bachelor’s degree and above	Employed	>5000	Urban

#### Beneficial experiences from the intervention

##### Gaining comprehensive health knowledge

Participants reported an enhanced understanding of hypertension, including its classification, causes (e.g., genetic factors and lifestyle), and associated risks. They appreciated practical tips, such as reducing salt intake, engaging in regular exercise, and monitoring blood pressure, which instilled confidence in managing their health.

##### Improvement in healthy behaviors

Participants adopted healthier habits, including regular exercise, dietary modifications to reduce salt and fat intake, and consistently monitoring their blood pressure using mobile applications. These behavioral changes fostered a sense of accomplishment and improved adherence to health guidelines.

##### Better blood pressure control and physical health

The structured intervention led to noticeable improvements in blood pressure stability and overall physical energy. Participants felt optimistic about sustaining these changes and received positive feedback from family members regarding their vitality and appearance.

##### Alleviation of psychological stress

Techniques such as mindfulness meditation and emotional journaling were effective in managing stress. Participants noted improvements in emotional regulation and a stronger sense of community through interactions with peers facing similar challenges.

#### Challenges and suggestions

##### Inconvenience in content delivery

Participants highlighted difficulties accessing intervention materials, which were distributed across various platforms (e.g., chat groups and public accounts). They suggested developing a centralized system, such as a dedicated app, for streamlined access.

##### Complexity of tracking tasks

The process of logging activities and sharing updates in multiple formats was considered cumbersome. Participants recommended simplified or integrated tools for monitoring and sharing progress to enhance usability.

##### Ineffective reminders

Participants found the existing reminder system insufficiently flexible. They proposed customized reminders that align with individual schedules and habits to improve adherence.

##### Limited peer interaction

Although group chats facilitated some exchanges, participants felt there was a lack of real-time and engaging communication. They suggested platforms featuring interactive elements, such as progress sharing or leaderboards, to boost motivation and community support.

## Discussion

This study evaluated the feasibility and preliminary effects of a physical literacy intervention program designed for young and middle-aged patients with hypertension. The findings demonstrate the program’s potential to improve physical literacy, increase participation in physical activity, and enhance associated health outcomes.

The intervention achieved high feasibility, with an 87.5% completion rate. Participants reported no adverse events, emphasizing the safety and acceptability of the program. The reasons for dropout, such as work-related conflicts and a foot injury, reflect the challenges of balancing health interventions with personal and professional obligations in this demographic. The demographic similarity between the intervention and control groups ensured baseline comparability, which strengthened the validity of the study outcomes.

While our findings demonstrate significant improvements in systolic blood pressure and physical literacy, it is important to contextualize these results within the broader literature, as not all exercise interventions have showed consistent benefits. For instance, a landmark randomized controlled trial by Blumenthal et al. (34) found that four months of aerobic exercise training failed to produce significant reductions in blood pressure compared with a control group in patients with mild hypertension. The discrepancy between these earlier findings and our positive results may be attributed to the multidimensional nature of our intervention. Unlike the isolated exercise prescriptions used in previous studies, our program integrated PL components, focusing not only on physical competence but also on psychological factors such as confidence, motivation, and knowledge. This holistic approach likely fostered better behavioral adherence and physiological adaptation, suggesting that combining physical activity with psychosocial support may be more effective than exercise alone in managing hypertension.

This interpretation is further supported by Gungor et al. (35), who found that supervised aerobic exercise was superior to home-based exercise in reducing 24-hour systolic blood pressure variability in postmenopausal women with hypertension. Their findings suggest that the level of supervision and structured support may be a critical determinant of exercise-related blood pressure benefits. In our study, the hybrid online–offline modality, weekly sessions, and WeChat-based monitoring provided a comparable level of structured support, which may partly explain the significant blood pressure improvements observed in the intervention group.

In our study, significant improvements in physical literacy were observed across all dimensions: cognition, emotion, physical competence, and behavior. This underscores the holistic approach of the intervention. These findings align with prior research, which emphasizes the importance of integrated strategies for fostering engagement in physical activity (36). Enhanced physical literacy was accompanied by notable improvements in physical activity levels, including increased weekly energy expenditure and moderate-intensity exercise. This suggests that the program effectively addressed participants’ barriers to physical activity. These findings are consistent with a recent systematic review and meta-analysis by Liu et al. (37), which demonstrated that mobile health interventions, including WeChat-based programs, significantly improved physical activity levels and blood pressure control in adults with hypertension. The authors highlighted that the effectiveness of such interventions may be attributed to their ability to deliver real-time feedback, facilitate self-monitoring, and provide ongoing social support—features that were also central to our WeChat-based intervention. This provides further evidence supporting the feasibility and potential scalability of mobile health–delivered physical literacy interventions in community settings.

The intervention group also experienced significant reductions in weight, BMI, and blood pressure, indicating that improved physical literacy can lead to tangible health benefits (38). These results highlight the critical role of physical literacy in managing hypertension through lifestyle changes. Participants reported behavioral changes, such as adopting regular exercise routines and healthier dietary habits, which were validated through qualitative feedback where they expressed increased confidence in managing their condition.

In addition to physical improvements, the intervention contributed to enhanced psychological well-being. Participants reported reduced stress levels and improved emotional regulation, facilitated by mindfulness meditation and peer support within the program (39). This reflects the program’s alignment with Antonovsky’s salutogenic model, which emphasizes the role of a sense of coherence in fostering health resilience. Social support also emerged as a vital component, promoting sustained engagement and a sense of community among participants.

Despite its success, the study identified areas for refinement. Participants expressed the need for streamlined content delivery and simplified activity tracking systems. Developing a dedicated app or centralized platform could help address these challenges, enhancing accessibility and user experience. Additionally, incorporating interactive elements, such as real-time communication and progress tracking, may further improve engagement and adherence (40).

The findings underscore the potential of physical literacy interventions as a non-pharmacological strategy for managing hypertension. By addressing cognitive, emotional, and behavioral dimensions, these programs empower patients to take ownership of their health. Future studies should explore the scalability and long-term effects of such interventions, particularly in diverse populations and settings. Incorporating objective measures, such as wearable fitness trackers, could provide deeper insights into behavioral patterns and the efficacy of the intervention.

## Strengths and limitations

Strengths of this study include its theoretical grounding in the salutogenic model, comprehensive mixed-methods evaluation, and high participant satisfaction. Several key limitations should also be acknowledged. First, the small sample size and single-site recruitment restrict the generalizability of study findings. Second, participants aged 18–59 years were analyzed as a single cohort; no age-stratified subgroup analysis was performed to compare intervention effects between younger and middle-aged subgroups. Third, quantitative analyses adopted complete-case analysis, which assumes data are missing completely at random (MCAR). Partial data from five participants lost-to-follow-up were not incorporated. Potential bias cannot be excluded if missingness was associated with study outcomes. Future larger-sample studies are warranted to explore differential intervention effects across age subgroups.

## Conclusion

This study shows that a structured physical literacy intervention can effectively increase physical activity, improve health outcomes, and enhance psychological well-being among young and middle-aged patients with hypertension. By tackling the challenges identified and utilizing technological solutions, such interventions could play a crucial role in managing chronic diseases.

## Data Availability

The raw data supporting the conclusions of this article will be made available by the authors, without undue reservation.
