# Supplementary material for: Effectiveness of a physical literacy intervention program for young and middle-aged adults with hypertension: a mixed-methods quasi-experimental study
Source: Front Endocrinol (Lausanne). 2026 Oct 20;17:1944805. doi: 10.3389/fendo.2026.1944805 (PMC13727378; doi:10.3389/fendo.2026.1944805)
Supplement: Supplementary file 1 [file SupplementaryFile1.docx]

**Intervention program for enhancing physical literacy among young and middle-aged adults with hypertension**

Brief note on program development: The initial draft was generated from systematic literature review, then iteratively revised through two-round expert Delphi consultation and patient focus-group discussion. This supplementary file shows the final week-by-week implementation protocol after revision. Raw Delphi questionnaires, expert rating datasets and focus-group transcripts are not included to avoid excessive file length.

| **Stage** | **Specific Contents** |
| --- | --- |
| **Preparation before intervention** | - **Establishing a medical team**: Define roles and responsibilities for each team member in managing patient health. All members receive relevant training before implementing the intervention.  - **Creating a WeChat public account**: Set up an account for centralized communication, resources dissemination, and interaction with participants. |
| **Intervention frequency and duration** | - The intervention spans 8 weeks, with one session per week.  - Sessions are delivered through a combination of 4 online and 4 offline activities. |
| **Week 1: Kickoff and building foundational knowledge** | - **Objectives**:  - Enhance participants’ understanding of hypertension.  - Establish awareness and interest in physical activity.  - Build self-management skills and an initial social support network.  - Improve sense of coherence.  - **Content**:  - **Introduction**: Explain the intervention’s purpose and benefits, followed by icebreaker activities to foster familiarity among participants.  - **Health education workshop**:  - Overview of hypertension (definition, causes, risk factors, symptoms, complications, and management).  - Benefits of physical activity for hypertension management and the role of physical literacy.  - Discuss connections between social support, self-efficacy, and hypertension control.  - **Assessment and goal setting**:  - One-on-one consultations to evaluate health status and lifestyle.  - Personalized blood pressure management goals.  - **Self-monitoring and blood pressure management**:  - Teach participants to use electronic blood pressure monitors and apps for tracking.  - Practical demonstrations and hands-on practice.  - **Resource distribution and social network formation**:  - Distribute comprehensive guides covering hypertension management and physical literacy.  - Establish family support groups and patient WeChat groups for peer interaction.  - **Delivery mode**: Offline at community centers.  - **Facilitators**: Researchers, healthcare professionals, and community staff. |
| **Week 2: Personalizing exercise plans and enhancing self-efficacy** | - **Objectives**:  - Deepen understanding of physical activity principles.  - Teach SMART goal-setting techniques.  - Improve self-efficacy through structured activities.  - **Content**:  - **Advanced physical activity knowledge**: Discuss suitable exercise types, intensity, and precautions.  - **SMART goal workshop**:  - Guide participants to set clear, measurable, achievable, relevant, and time-bound goals.  - Interactive exercises to ensure comprehension and application of SMART principles.  - **Physical literacy assessment**: Administer validated tools to evaluate physical literacy and tailor future activities.  - **Ongoing support**: Regular monitoring via WeChat, with personalized feedback and family involvement.  - **Delivery mode**: Offline at community centers.  - **Facilitators**: Researchers and community staff. |
| **Week 3: Comprehensive physical activity and psychological management** | - **Objectives**:  - Enhance physical activity skills.  - Strengthen physical and psychological capacity.  - Reinforce comprehensibility and manageability as aspects of sense of coherence.  - **Content**:  - **Practical physical activities**:  - Warm-ups, balance training, and strength exercises (e.g., bodyweight squats, resistance band stretches).  - Flexible options for activities like walking, dancing, and traditional exercises (e.g., Baduanjin).  - **Psychological journaling**: Introduce reflective practices to enhance self-awareness and track progress.  - **Sustained support**: Monitor progress via family feedback and provide regular guidance.  - **Delivery mode**: Online via WeChat.  - **Facilitators**: Researchers and healthcare professionals. |
| **Week 4: Advanced physical and psychological strategies** | - **Objectives**:  - Master advanced exercise techniques and mindfulness practices.  - Foster family engagement in health activities.  - **Content**:  - **Advanced physical activities**: Focus on flexibility, coordination, and traditional martial arts (e.g., Twelve‑form boxing).  - **Mindfulness and immersion**: Teach mindfulness techniques for emotional regulation and integration into physical activities.  - **Family health workshops**: Promote shared exercise experiences and enhance family support dynamics.  - **Delivery mode**: Online via WeChat.  - **Facilitators**: Researchers and healthcare professionals. |
| **Week 5: Sustaining healthy behaviors** | - **Objectives**:  - Strengthen adherence to healthy behaviors.  - Establish a robust social support network.  - Achieve comprehensive health improvements.  - **Content**:  - **Behavioral feedback and reinforcement**: Collect feedback on progress and provide tailored recommendations.  - **Physical activity practice**: Options include cycling, dance, and advanced Qigong techniques (e.g., Yijin Jing).  - **Long-term strategy development**: Equip participants with tools and skills for sustained health behavior change.  - **Delivery mode**: Online via WeChat.  - **Facilitators**: Researchers and community staff. |
| **Week 6: Integrated physical activity and emotional regulation training** | - **Objectives**:  - Enhance the participants’ ability to manage emotions through exercise.  - Strengthen participants’ understanding of the relationship between physical activity and psychological well-being.  - Promote the incorporation of emotional regulation strategies into daily routines.  - **Content**:  - **Physical activity practices**:  - **Warm-up**: Include dynamic stretching and light aerobic exercises, such as arm swings and side steps.  - **Core training**:  - Balance exercises: Single-leg stance and heel-to-toe walking.  - Flexibility and coordination drills: Gentle yoga poses (e.g., Warrior Pose and Downward Dog).  - **Activity options**:  - Option 1: Brisk walking combined with breathing exercises.  - Option 2: Chinese traditional martial arts, focusing on advanced movements from Twelve‑form boxing. - Option 3: Gentle aerobic dance or functional fitness routines.  - **Cool-down**: Relaxation stretches, focusing on deep breathing and muscle relaxation.  - **Emotional regulation techniques**:  - Introduce simple mindfulness exercises and progressive muscle relaxation.  - Guide participants in journaling emotional states and their implications for physical activity adherence.  - **Social support enhancement**:  - Facilitate group discussions in WeChat groups to share experiences and encourage peer support.  - Encourage participants to involve family members in joint activities for mutual motivation.  - **Delivery mode**: Online via WeChat.  - **Facilitators**: Researchers and community healthcare providers. |
| **Week 7: Advanced integration of physical and psychological well-being** | - **Objectives**:  - Encourage the sustainable adoption of physical activity and healthy habits.  - Advance participants’ skills in integrating physical literacy into daily life.  - Strengthen family and community roles in promoting health behaviors.  - **Content**:  - **Advanced physical activities**:  - **Warm-up**: Dynamic movements and joint activation exercises.  - **Core training**:  - Strength and endurance exercises (e.g., bodyweight lunges, plank holds).  - Balance enhancement with closed-eye single-leg stance.  - **Activity options**:  - Option 1: Jogging with alternating slow and fast intervals.  - Option 2: Advanced Taijiquan for relaxation and balance.  - Option 3: Comprehensive Qigong routines (e.g., Yijin Jing).  - **Cool-down**: Stretching routines emphasizing joint flexibility and relaxation.  - **Family and community engagement**:  - Conduct family health promotion workshops.  - Facilitate small-group challenges and collaborative physical activities to strengthen bonds.  - **Behavioral reinforcement**:  - Encourage participants to reflect on health progress and personal achievements.  - Provide ongoing feedback and positive reinforcement to sustain motivation.  - **Delivery mode**: Offline at community centers.  - **Facilitators**: Researchers and trained facilitators. |
| **Week 8: Closing and future planning** | - **Objectives**:  - Evaluate the outcomes of the intervention program.  - Develop long-term health action plans with participants.  - Celebrate achievements and motivate sustained lifestyle changes.  - **Content**:  - **program evaluation**:  - Reassess physical literacy levels using validated tools.  - Collect participant feedback through structured surveys and group discussions.  - **Long-term planning**:  - Guide participants in developing personalized health and physical activity plans.  - Provide resources and contacts for continued support (e.g., community classes, healthcare services).  - **Celebration of success**:  - Host a group session to recognize participant achievements.  - Share individual progress stories to inspire continued efforts.  - **Social network consolidation**:  - Strengthen WeChat group connections for peer support.  - Encourage ongoing engagement through shared health goals and activities.  - **Delivery mode**: Offline at community centers.  - **Facilitators**: Researchers, healthcare professionals, and community staff. |
