# Supplementary material for: Effectiveness of a physical literacy intervention program for young and middle-aged adults with hypertension: a mixed-methods quasi-experimental study
Source: Front Endocrinol (Lausanne). 2026 Oct 20;17:1944805. doi: 10.3389/fendo.2026.1944805 (PMC13727378; doi:10.3389/fendo.2026.1944805)
Supplement: Supplementary file 2 [file SupplementaryFile2.docx]

**Semi-structured interview guide**

1. What is your overall evaluation of this entire intervention program?

2. Do you consider the hypertension‑related knowledge you gained from the program helpful for your daily life? Could you share one or two practical examples of how you have put this knowledge into practice?

3. In your view, how important is the role of physical activity in hypertension management? Has the program motivated you to take part in physical exercise more regularly?

4. Have your exercise habits changed since you took part in this intervention? Are you able to maintain a consistent volume of physical activity on a daily basis?

5. Have you perceived any reduction in psychological stress following participation in the intervention program? Have you practised any of the relaxation techniques recommended throughout the program?

6. Did you find peer support and peer interaction beneficial during the intervention? Has this form of support enhanced your adherence to the program?

7. Following this two‑month intervention, have you noticed any improvements in your blood pressure? Could you describe your subjective experiences or share relevant blood‑pressure‑related data where available?

8. Do you have any suggestions for revising this intervention program, particularly with respect to its content and delivery modalities?
